# Factors Influencing Medical Personnel to Work in Primary Health Care Institutions: An Extended Theory of Planned Behavior

**DOI:** 10.3390/ijerph19052785

**Published:** 2022-02-27

**Authors:** Huanhuan Jia, Jianxing Yu, Tianyu Feng, Liangwen Ning, Peng Cao, Panpan Shang, Shang Gao, Xihe Yu

**Affiliations:** 1 School of Public Health, Jilin University, Changchun 130012, China; jhh_1994@163.com (H.J.); yjxjlu@163.com (J.Y.); fengty21@mails.jlu.edu.cn (T.F.); cppengcao@163.com (P.C.); shangpan12306@126.com (P.S.); sobermz@163.com (S.G.); 2 School of Public Administration, Jilin University, Changchun 130012, China; ningliangwen@163.com

**Keywords:** primary health care, medical personnel, theory of plan theory, implicit theory, lexical approach, China

## Abstract

In China, the primary health care institutions (PHCIs) have difficulty winning the trust of residents because of the shortage of medical personnel and the low level of skills. The government has advocated encouraging qualified doctors to work in PHCIs, but no obvious results are achieved. Based on the theory of planned behavior (TPB), this paper explores the factors affecting medical personnel seeking employment in PHCIs and then putting forward measures to improve the human resources construction of PHCIs. A three-stage survey was conducted to obtain the factors and a structural equation modeling (SEM) was applied to examine the relationship of the factors. We found that the factors affecting medical personnel to work in PHCIs include the specific conditions and work contents of PHCIs, as well as the family life and regional factors. Besides, there is a significant correlation and indirectness between these factors. Therefore, targeted measures can be proposed to improve the intention of medical personnel seeking employment in PHCIs. This study provides theoretical support for encouraging medical personnel to work in PHCIs and improving the primary health care system.

## 1. Background

Primary health care (PHC) is the most inclusive, equitable, cost-effective, and efficient approach to enhancing people’s physical and mental health, as well as their social well-being, and it is the cornerstone of a sustainable health system that can achieve universal health coverage, reach sustainable health-related development goals, and promote health security [1]. As a country with a large population and vast territory, China’s primary health care services play a vital role in improving the fairness and accessibility of health services in general. Primary health care services are provided by primary health care institutions (PHCIs), mainly including community health service centers and township hospitals [2]. However, since the 20th century, the operation of PHCIs has been stagnant. Due to the scarcity of medical resources, insufficient funds, the absence of the newest medical technology [3], and few professional and qualified medical staff [4,5], PHCIs have had difficulty winning the trust of residents, and residents’ willingness to seek medical treatment in PHCIs is getting worse, with many showing a strong preference for high-level hospitals [6]. Therefore, poor-quality health care delivered by providers in PHCIs is a main challenge to China’s achievement of UHC, and improving the primary health care system remains a central part of China’s health-care reform [7].

In 2009, China began a new medical reform, in which the improvement of the PHC system was one of the five key reforms [8], and the Chinese government increased its focus on the construction of PHCIs. In addition, the hierarchical medical system was proposed as a means to reduce the burden of health expenditure and promote the development of PHCIs by classifying patients according to the severity of disease and difficulty of treatment, with the aim of distributing responsibility for the treatment of different diseases to medical institutions of different levels. At the same time, the policy envisages transferring the general outpatient services, rehabilitation, and nursing services that had previously been undertaken by large and medium-sized medical institutions to PHCIs in order to establish mechanisms for first visits to PHCIs and bidirectional referral [9]. This shift demonstrates the important responsibilities and the central role of PHCIs in the health system, which require PHCIs to be equipped with adequate numbers of skilled human resources to promote health. As a result, various types of training and practical work have been carried out to train PHCI workers [10], and the government has advocated encouraging qualified doctors to work in PHCIs [11]. However, the government has not offered specific measures to encourage qualified doctors to work in PHCIs, and the few measures for talent introduction issued by local governments or PHCIs have not achieved obvious results. The lack of human resources in PHCIs has led to a failure of responsibility by the “Gatekeeper” of residents’ health, and this situation has greatly hindered the construction of primary medical service systems and the implementation of hierarchical medical systems. 

Therefore, it is crucial to identifying the influencing factors of medical staff seeking employment in PHCIs and formulating measures to guide medical staff to work in PHCIs.

## 2. Theoretical Foundation

Previous researches on human resource in the healthcare sector have shown that the demand for medical personnel is rapid growth [12] and the capacity of human resource managers [13], the remuneration, career development, and succession plan [14] are important factors in the development of human resources in health. However, these research or talent acquisition measures were based solely on the perspective of local government health departments or medical institutions, focused on individual health care workers and with less research on PHCIs. There was a lack of comprehensive exploration of and research into the influencing factors from the perspective of medical personnel, a deficiency which may lead to a mismatch between recruitment measures of PHCIs and the needs of medical personnel. Therefore, this study changes the medical personnel from passive recipients of policy to participants in policy formulation and excavates the factors influencing PHCI employment from the point of view of medical personnel. The study then formulates measures for the introduction of talent according to these factors.

**Figure 1:**
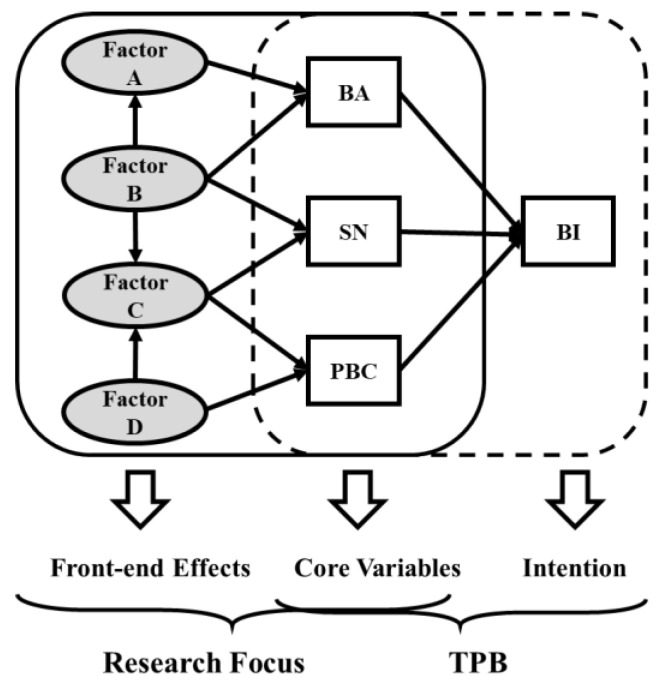
The hypothetical structural equation model. Note: BA, behavioral attitudes; SN, subjective norms; PBC, perceived behavioral control; BI, behavior intention; TPB, theory of planned behavior.

The theory of planned behavior (TPB), one of the most influential behavioral models in social psychology, proposes that behavior intention (BI) is an accurate predictor of behavior [15]; that is, the key proximal determinant of behavior is the person’s intention to perform the behavior [16]. BI is based on three core variables: behavioral attitudes (BA), subjective norms (SN), and perceived behavioral control (PBC). BA refers to an individual’s positive or negative feeling about performing a target behavior. SN represents the social influences on an individual’s engagement in a particular behavior. PBC refers to an individual’s perceived control in performing a behavior [17]. According to PBC, intentions will be stronger when individuals and their reference groups positively evaluate a given behavior and realize that nothing can prevent them from engaging in that behavior [18]. TPB has wide applications, in cases such as willingness to use healthier travel modes [19], drinking behavior [20], and drug adherence [21], but few studies have explored what influences these three factors. As depicted in Figure 1, BA, SN, and PBC are closely related to BI, but fewer studies have analyzed the front-end effects of these three core variables. For different groups of people and target behaviors, the factors that influence the three variables are different; that is, the content, number, and relationship of factors A, B, C, and D will change. By understanding the factors that influence a target behavior among a particular group of people and their relationship to BA, SN, and PBC, specific measures can be put forward to improve BI.

Therefore, this study not only applies TPB to an exploration of the influence of BA, SN, and PBC on the BI of medical personnel seeking employment in PHCIs but also explores the factors affecting BA, SN, and PBC. On the one hand, a few studies have explored the influencing factors of BA, SN, and PBC. On the other hand, this study was conducted from a new perspective of medical personnel. As such, few studies can be referenced. Therefore, this study needs to understand the factors first and then explore the relationship between these factors and TPB.

Implicit theory refers to people’s views on the concept, structure, and development of certain psychological features, which are formed in the background of daily life and work and exist in some form within the individual’s mind; implicit theory can accurately and fully reflect the ideas in people’s minds [22]. Its role in the organization and interpretation of information is increasingly accepted by cognitive and social psychologists [23]; in recent years, its scope has expanded to include self-regulation learning [24], psychological attributes [25], and leadership effectiveness [26]. The lexical approach is one of the methods of implicit theory, which is based on the hypothesis that the most important differences in people’s daily communication will eventually be encoded in their language [27,28]. The main significance of this approach is that it provides a research strategy aimed at identifying a set of relatively small, largely independent axes along which people typically exhibiting behavioral differences [29]. The lexical approach has been successfully applied in many fields of psychology, such as values [30], personality psychology [31], and human-computer interaction [32]. The argument here is that, in the context of China’s medical reform, medical personnel are exposed to information about the current situation and talent introduction of PHCIs in the course of their lives and work, which is encoded in minds and languages. Therefore, the lexical approach can be applied to explore the attitudes of medical personnel toward PHCIs. On this basis, the lexical approach was applied to collect the factors that affect medical personnel seeking employment in PHCIs, and to explore the relationship between these factors and TPB in order to expand the front-end influence of TPB and to put forward measures to encourage medical personnel to work in PHCIs.

Generally speaking, based on TPB, this paper uses the implicit theory to collect and explore the factors that affect medical personnel seeking employment in PHCIs, and puts forward measures to improving the human resources construction of PHCIs.

## 3. Methods

### 3.1. Study Design and Participants

In accordance with the paradigm of the lexicology hypothesis [33,34], the collection of factors consists of two processes. The first stage is a presurvey aimed at collecting the items that lead medical personnel to work in PHCIs, for which subjects were randomly selected from two hospitals in a certain area. The second stage is to classify the items collected, that is, to reduce the items collected in the first stage to representative factors by factor analysis. In addition, a third stage is added to analyze the relationship between these factors and BA, SN, and PBC.

Pursuant to discussion with experts in health services, hospital management and the head of the health administration department, the subjects of the second and third stages consisted of a set of medical personnel from Jilin Province that had been obtained by stratified random sampling. First, all areas of Jilin Province were divided into cities and counties, and hospitals were divided into urban public hospitals and county-level public hospitals. Because urban public hospitals were highly clustered, they were stratified by region, type of institution and level and then randomly selected at a rate of 1/4. Because of the dispersion of different counties, the People’s Hospital and the traditional Chinese medicine hospital of each county were selected as sample hospitals. A total of 109 hospitals were selected, including 29 urban public hospitals and 80 county-level public hospitals. Through convenient sampling, 30 doctors and nurses from hospitals were selected as survey subjects. A total of 3270 medical staff participated in the study. It should be noted that the hospitals selected in the second and third stages were the same, but because of random selection, the medical personnel may be different.

The procedure was approved by the Medical Ethics Committee of the author’s institute (No.20181102). The participants received a full explanation of the purpose of the survey, were told that the information collected was only for the study, and could withdraw from the study at any time.

### 3.2. Measures

In the first stage, the survey was managed by the data collection service of China’s leading online survey website, and the questionnaire was open-ended. Respondents were asked to answer the following question: *“If a PHCI is recruiting medical personnel, what are the factors that influence you to go to the PHCI for employment? Please write these factors in simple words or phrases”.* At the same time, the questionnaire also included the respondents’ sociodemographic characteristics, including gender, age, marriage status, education and majors. Several measures were taken in the questionnaire design and data cleaning to ensure data quality. For example, screening questions were set to ensure that the answers came from the right respondents, including the following: “*Are you a regular employee of the hospital?*”, “*What is your department*?”, and “*What is your major?*”. At the same time, IP confirmation (that is, using the same computer and geographical restrictions to prevent a respondent from filling in the questionnaire multiple times or to prevent people from other areas from filling in the questionnaire), answer time audit, and empty item review were adopted to ensure data quality.

The items collected in the first stage were used to compile a questionnaire for the second stage. The questions at this stage were introduced by the following: “*If a PHCI is recruiting medical personnel, please evaluate the importance of the following factors in terms of their influence on your choice to seek employment at the PHCI based on your actual situation*”, and items were graded with a range from 1 (very unimportant) to 5 (very important). Similarly, the questionnaire also included the sociodemographic characteristics of the respondents (gender, age, educational background, major, etc.,).

The third stage consisted of three parts. The first part addressed sociodemographic characteristics (gender, age, educational background, major, etc.,). The second part dealt with the representative factors that emerged after data analysis of the second stage, and the measurement method was the same as that of the second stage. The third part focused on the measurement of TPB. In the third part, items were taken from the previous study of TPB, and the wording was modified according to our study. Then, the expert consultation method was used to demonstrate and revise the questionnaire. Ten senior experts in the fields of health administration and medical education were invited to review the items in terms of scientificity, comprehensiveness, representativeness, operability, and importance. After 3–4 rounds of expert opinion collection and feedback, the final version of the questionnaire was obtained. Finally, a preliminary investigation of 50 medical personnel was conducted to evaluate and revise items to ensure the measurability of the questionnaire. In addition, the purpose of this study was to determine which factors would increase the intention of medical personnel to seek employment in PHCIs, so the part of the study concerned with TPB was assessed using a positive rating scale, namely, a Likert scale of five, ranging from 1 (strongly disagree) to 5 (strongly agree).

The items of TPB are shows in Table 1.

### 3.3. Data Analysis

According to the following principles, the items collected in the first stage were cleaned up and organized without changing the meaning: (1) Removal of modifiers and extraction of key information; (2) synonym combination; (3) splitting of combined concepts; (4) deletion of words that were obviously irrelevant to the research. In addition, two people categorized the vocabulary separately, compared the results, and decided on the final results in consultation with an expert group.

In the second stage, SPSS software (version 23.0, IBM Corporation, Armonk, NY, USA) was used to perform exploratory factor analysis (EFA) in order to obtain concise and representative factors, and principal component analysis (PCA) and variance rotation (VR) were also used to perform EFA. In addition, based on previous research, a hypothetical path model of the relationship between these factors and TPB was proposed. 

For the third stage, the hypothesis path model was tested using AMOS software (Version 24.0, IBM Corporation, Armonk, NY, USA) with the maximum likelihood, and the bootstrap was used to test the potential mediating effects. Finally, the direct, indirect, and total effects of each factor on BA, SN, and PBC were estimated. All statistical tests were two-sided with the level of significance set at 0.05.

## 4. Results

### 4.1. Factors Collection

A total of 546 medical personnel participated in the first stage, and 22 personnel working in preventive medicine, finance, and administration were deleted, resulting in 524 complete and valid responses, with an effective response rate of 95.97%. A majority of participants were female (74.81%), had a mean age was 26.41 years, had a bachelor’s degree (67.40%), and majored in clinical medicine (86.07%). After word cleansing and organization, all words were eventually recoded to 102 items. Frequency analysis showed that the frequency of 55 factors was less than 4; that is, those factors were mentioned by less than 1% of medical personnel, so the items were deleted due to lack of representation. The remaining 47 items were regularized for the preparation of the second stage.

### 4.2. Exploratory Factorial Analysis and Research Hypotheses

The EFA results showed that the Kaiser-Meyer-Olkin (KMO) was 0.981, and Bartlett’s test was significant (*p* < 0.001), which indicated that the data were suitable for EFA. The first results showed that six factors with eigenvalues greater than 1 were rotated, which accounted for 69.954% of the variance. However, there are some items with factor loads less than 0.45 or loads more than 0.4 in two dimensions, so they are deleted at each step. Finally, seven items were deleted, and the remaining 40 items were rotated into 6 factors, accounting for 71.597% of the variance. Table 2 details the results of the final EFA and the nomenclature of each factor.

Remuneration and development (RD) includes 8 items, such as wages, work subsidies, performance assessments, and position promotion, which reflect the concerns of medical personnel about remuneration and individual development opportunities while working in PHCIs. Sense of gain (SG) includes 4 items, including fulfilling personal value, job-related well-being, professional pride and basic living security, reflecting the emotional needs of medical personnel, which are psychological category rather than economic category. For medical personnel, training is more difficult and the initial investment is higher than other professional groups [35], so they usually pay great attention to remuneration and individual development opportunities. In addition, because of the sacred and noble nature of medicine, medical personnel tend to pay more attention to work-related emotional reactions, such as professional pride and personal value, than other occupational groups. Previous studies pointed out that the professional pride and personal value of medical personnel were closely related to their job quality, job satisfaction, and turnover intention [36,37]. At the same time, job-related well-being was the emotional reaction of employees and has been identified as an important area to attract and retain employees [38]. According to the theory of expectation-value, attitude is a kind of presupposed position that a person will like or dislike a certain object for an ongoing period or a person’s positive and negative evaluation of performing a certain behavior [39]. In our study, attitude reflects whether medical personnel have a positive attitude toward seeking employment in PHCIs. At the same time, based on the results of the first stage, we proposed that attitude was reflected in the remuneration, individual development, and emotional reactions of medical personnel. Therefore, we propose that remuneration and development and sense of gain can positively influence the attitude of medical staff to seek employment in PHCIs. At the same time, previous studies have pointed out that remuneration and development are important factors that affect the job satisfaction and work enthusiasm of community health workers [40]. Therefore, we assume that remuneration and development can not only directly affect attitude but also have an indirect impact on attitude toward seeking employment in PHCIs through sense of gain.

Therefore, we hypothesize as follows:

**Hypothesis** **1** **(H1):** RD has a positive effect on BA.

**Hypothesis** **2** **(H2):** SG has a positive effect on BA.

**Hypothesis** **2a** **(H2a):** RD has a positive indirect effect on BA through SG.

Job responsibility (JR), which reflects medical personnel’s attention to specific work position and work content, contains four items: namely, workload, working stress, working intensity, and working hours [39]. Related research also notes that job responsibility is an important factor that affects the satisfaction and turnover intention of medical personnel in PHCIs. Perceptual behavioral control (PBC) reflects the degree of control (or mastery) that an individual feels when he expects to engage in a particular behavior. For medical personnel, the job responsibility of PHCIs is within their grasp, and their technical ability, work experience, and work style can be sufficient for job responsibility, which is conducive to the increase of PBC and can thus increase intention to seek employment in PHCIs. Family support (FS) includes four items: spouse, parents, children, and house, which reflects the influence of people or things that are important to the career choices of medical personnel. The study points out that family support can relieve work stress and protect medical personnel from negative effects such as job burnout [41], while work-family conflict has a great influence on the high turnover rate of medical personnel [42]. The relevance of the subjective norm (SN) to PBC stems from the pressure generated by important people or groups around the individual towards the performance (or lack of performance) of a certain action. We found that FS and SN coincide. Therefore, we propose that the SN that affects whether medical personnel seek employment in PHCIs mainly comes from familial pressure. At the same time, changes in remuneration and development, as well as changes in job responsibilities, will inevitably effect changes in the living conditions of the family, which, in turn, will affect the pressure of the family on individuals to engage in a certain behavior, that is, subjective norms.

Therefore, we hypothesize as follows:

**Hypothesis** **3** **(H3):** JR has a positive effect on SN.

**Hypothesis** **4** **(H4):** JS has a positive effect on PBC.

**Hypothesis** **4a** **(H4a):** RD has a positive indirect effect on SN through FS.

**Hypothesis** **4b** **(H4b):** JR has a positive indirect effect on SN through JS.

The remaining items were rotated to two factors, among which 13 items, such as department setting, specialist construction, regulatory regime and software and hardware facilities, reflected internal organizational development (IOD), while the remaining 7 items, such as location, economics, environment and transportation, reflect the condition of the city where the PHCI is located (CCPL). After analysis, we found that these two factors and the three core dimensions of TPB are not obviously related directly, whereas there are obvious links between these two factors and other factors.

The work of medical personnel is influenced by factors such as organizational and management system, department construction and organizational culture, which directly affect work positions, specific work contents, remuneration, and individual development. Additionally, emotional reactions have an impact that cannot be ignored. For the external environment of PHCIs, regional economic level, culture and urban development can not only affect income level and development opportunities but can also have an impact on emotional reactions; at the same time, these factors have an impact on the life and work of family members.

Therefore, we hypothesize as follows:

**Hypothesis** **5a** **(H5a):** IOD has a positive indirect effect on BA through SG.

**Hypothesis** **5b** **(H5b):** IOD has a positive indirect effect on BA through RD.

**Hypothesis** **5c** **(H5c):** IOD has a positive indirect effect on PBC through JR.

**Hypothesis** **6a** **(H6a):** CCPL has a positive indirect effect on BA through SG.

**Hypothesis** **6b** **(H6b):** CCPL has a positive indirect effect on BA through RD.

**Hypothesis** **6c** **(H6c):** CCPL has a positive indirect effect on SN through FS

### 4.3. Model Test

Confirmatory factor analysis (CFA) was applied to test the reliability and validity of our measurement tools. The results showed that Cronbach’s alpha values ranged from 0.777 to 0.961, indicating high reliability. Composite reliability (CR) values were greater than 0.9, indicating high internal consistency among the constructs. At the same time, the average variance extraction value (AVE) of each construct was above 0.7, and the standardized factor loading of each item was above 0.7, which verified good convergence validity [43,44]. Therefore, the measuring tools have good reliability and validity. The results are shown in Table 3.

**Figure 2:**
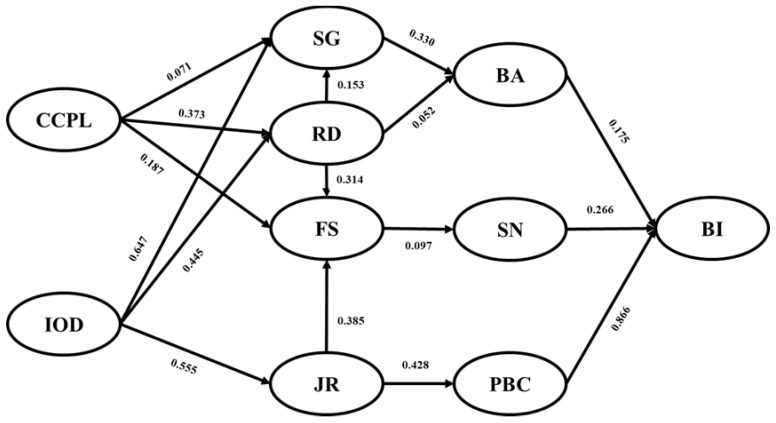
The results of the structural equation model. Note: SG, sense of gain; RD, remuneration and development; IOD, internal organization development; CCPL, condition of the city where the PHCI is located; JR, job responsibilities; FS, family support, BA, behavior attitude; SN subjective norm; PBC, perceived behavioral control; BI, behavior intention.

The hypothesis test uses maximum likelihood to fit the data and calculates a T value for each path to study the model’s hypothesis. SG (*β* = 0.330; *p* < 0.001) and RD (*β* = 0.052; *p* = 0.002) had a significant positive effect on BA. FS (*β* = 0.097; *p* < 0.001) had a significant positive effect on SN, and JR (*β* = 0.428; *p* < 0.001) had a significant positive effect on PBC, so H3 and H4 were supported. The results of the structural equation model are demonstrated in Figure 2.

At the same time, bootstrapping was applied to explore the mediating effect in the hypothesis, and a 95% confidence interval for the indirect effects was obtained with 5000 bootstrap resamples. The results showed that the mediating effect of SG on RD and BA was significant, so H2a was supported, and RD and JR significantly affected SN and PBC through FS, respectively, so H4a and H4b were supported. The effects of IOD and CCPL on BA through SG and RD were significant, the effect of CCPL on SN through FS was significant, and the effect of IOD on PBC through JR was significant, so H5a, H5b, and H5c and H6a, H6b, and H6Cc were also supported. Table 4 shows the direct and indirect effects of each factor on the three core variables of TPB.

## 5. Discussion

Based on TPB and implicit theory, this study explores the factors that affect medical personnel seeking employment in PHCIs from the point of view of medical personnel and extends the application of TPB in the context of encouraging medical personnel to work in PHCIs. We found that the factors can be summarized into 6 factors, including condition of the city where the PHCI is located (CCPL), remuneration and development (RD), internal organization development (IOD), job responsibilities (JR), family support (FS) and sense of gain (SG). In addition, these factors can directly or indirectly affect the behavioral attitude (BA), subjective norms (SN), and perceptual behavior control (PBC) of TPB and then have an impact on the intention of medical personnel to seek employment in PHCIs.

PBC has the highest correlation with BI, which is similar to previous research on TPB [45,46], which reflects the assessment of the individual’s ability before a behavior is performed. For PHCIs, medical personnel provides both basic public health services and health care services [47]. At the same time, in China’s medical reform plan, PHCIs take on the responsibilities of a “first visit” for diseases and the care of patients in stable or convalescent periods, so PHCI providers take on a great deal of work and play a vitally important social role. Previous research has also pointed out that the workload and pressure on PHCIs are important reasons for the departure of medical personnel [48]. Therefore, the intention of medical personnel to seek employment in PHCIs largely depends on an assessment of individual capabilities and the job responsibilities within their control. In addition, this study shows that job responsibility is reflected in workload, stress, intensity, and time rather than in work difficulty and technical level. PHCIs provide primary health care, so most of the services are less technically difficult than services provided by hospitals, so medical personnel believe that they are competent and pay more attention to the amount of work. Therefore, we propose that balancing or appropriately reducing the work pressure and workload of medical personnel in PHCIs is an important measure for encouraging medical personnel to work in PHCIs.

The results show that family members are an important social group that influences the intention of medical personnel to work in PHCIs, which is consistent with the Chinese tradition of attaching importance to family culture. Previous studies [49,50] have shown that most people look to family members for advice or take into account their lives and work when choosing a career, and family members are also important to the work of health care professionals. Therefore, we propose considering the impact of changes in the work of medical personnel on the lives and work of their family members when encouraging medical staff to work in PHCIs and the adoption of appropriate work arrangements and compensation policies to encourage family members to take a positive attitude toward the changes, thereby improving the intention of medical personnel to seek employment.

RD and SG reflect medical personnel’s realistic and emotional needs for work, respectively. The results show that RD and SG can positively influence BI, and RD has a significant positive and indirect effect on BA through SG. This fact not only reflects the common needs of job seekers but is also closely related to the working status of medical personnel in PHCIs. Previous studies [51,52,53] have pointed out that low wages and insufficient development opportunities have resulted in low satisfaction, job burnout, increased turnover of medical personnel in PHCIs and difficulties in talent introduction. Under China’s medical system, PHCIs bear the important responsibility of providing basic medical and health services and promoting health coverage for residents, whereas their wages are significantly lower than those of medical personnel in hospitals [54]. In addition, the income of Chinese doctors stands in sharp contrast to the United States and other western countries, where physicians are recognized as a high-income profession and the return on professional education for physicians is high [55]. Moreover, income level and development opportunity are significant predictors of general practitioners’ professional identity [56]. Therefore, medical personnel pay more attention to remuneration when they seek employment in PHCIs. We propose that appropriate subsidies and increased training and learning opportunities are conducive to increasing the intention of medical personnel seeking employment in PHCIs. In addition, awareness of PHCIs’ services among medical personnel should be enhanced to improve their sense of participation and thus their motivation to seek employment in PHCIs.

The effects of internal construction and location on BA, SN, and PBC are indirect and significant. First, the construction of PHCIs, such as management systems, human resource allocation and software and hardware facilities, directly affects work arrangement, wages and individual development; at the same time, organizational culture and interpersonal relationships are vitally important for the emotional needs of medical personnel. Second, the economy, culture, and development of a region have an imperceptible influence on the living standard and emotional life of all the people in the region, which inevitably connects with the flow of people due to work. Therefore, we propose to improve the construction of PHCIs through measures such as equipping them with software and hardware facilities, formulating appropriate management systems and reasonably arranging work, and providing a high-quality working environment for medical personnel. In addition, through measures such as publicity and guidance, we propose to create a social environment that respects doctors, attaches importance to medical treatment, and advocates medicine.

Several limitations of the current study should be acknowledged. First, the cross-sectional study was unable to determine the causal relationship between factors. Second, the study was conducted in only one province in China, while the differences between regions of China are large, which may limit the generalization of the findings. Third, changes in respondents between the second and third stages may skew the results, but we have fully communicated with experts and local government leaders to ensure that the sample accurately reflects the overall situation. In the further research, first, the sample will be gradually expanded and samples from different regions of China will be selected for analysis and comparison. Second, a longitudinal study will be conducted to observe whether the willingness and influencing factors of medical personnel seeking employment in PHCIs will change with the deepening of health reform and the implementation of policies.

## 6. Conclusions

This study extends TPB and discusses the factors that affect medical personnel seeking employment in PHCIs based on implicit theory. We found that (1) the main factors that affect medical personnel seeking employment in PHCIs include sense of gain, remuneration and development, family support, job responsibility, internal organization development, and condition of the city where the PHCIs are located. (2) The improvement of wages, development opportunities, and sense of gain can strengthen the behavior attitude of medical personnel seeking employment in PHCIs. (3) Family factors and job responsibilities have positive effects on the subjective norms and perceptual behavior control of medical personnel seeking employment in PHCIs. (4) The internal and external environment of PHCIs has a significant indirect effect on attitudes, subjective norms, and perceptual behavior control. This study is beneficial to the implementation of the government’s health care reform policy, the development of PHCIs, and the protection of the rights and interests of medical personnel, which are ultimately beneficial to the improvement of PHC and the protection of the health of the population.

## Figures and Tables

**Table 1:** The items of TPB.

Factors	Items
Behavior Attitude (BA)	BA1. Working in PHCIs is positive for medical reform.
	BA2. Working in PHCIs is meaningful.
	BA3. Working in PHCIs would improve my quality of life.
	BA4. Working in PHCIs would take the pressure off my life.
Subjective Norm (SN)	SN1. Most of the people who matter to me support my working in PHCIs.
	SN2. Some of my colleagues have started working in PHCIs.
	SN3. The social function of PHCIs inspires me to work in a PHCI.
Perceived Behavioral Control (PBC)	PBC1. My work style can be competent enough for the work of PHCIs
	PBC2. I have enough medical experience to work in PHCIs.
	PBC3. My technical ability is sufficient for the work of PHCIs.
	PBC4. If I wanted to, I could easily get a job in a PHCI.
Behavior Intention (BI)	BI1. I would like to know more about the work of PHCIs.
	BI2. I will focus on the development of PHCIs.
	BI3. If the development of PHCIs improves, I may consider working in a PHCI.
	BI4. If there were a suitable PHCI, I would work there.

**Table 2:** The results of the final EFA.

Factors	Items	Component					
		1	2	3	4	5	6
Sense of Gain	Fulfilling Personal Value	0.747					
	Job-Related Well-being	0.694					
	Professional Pride	0.692					
	Basic Living Security	0.501					
Internal Organization Development	Knowledge Level	0.748					
	Department Setting	0.705					
	Specialist Construction	0.705					
	Teaching and scientific research	0.692					
	Learning Atmosphere	0.691					
	Personnel Quality	0.673					
	Regulatory regime	0.671					
	Software and Hardware Facilities	0.668					
	Human resource allocation	0.663					
	Hospital Culture	0.653					
	Services Scope	0.648					
	Leading and Administrative Capacity	0.605					
	Internal interpersonal relationship	0.472					
Remuneration and Development	Wage		0.786				
	Working Subsidy		0.782				
	Medical Insurance		0.780				
	Social Insurance and Housing Accumulation Fund		0.720				
	Working Bonus		0.691				
	Holidays Arrangements		0.671				
	Performance Assessment		0.666				
	Position Promotion		0.498				
Condition of the City Where the PHCI Is Located	Location			0.728			
	Culture and Customs			0.693			
	Development			0.692			
	Economics			0.691			
	Environment			0.668			
	Transportation			0.640			
	Local reputation of the PHCI			0.624			
Job Responsibilities	Workload				0.853		
	Working Stress				0.830		
	Working Intensity				0.826		
	Working Hours				0.770		
Family Support	Spouse					0.795	
	House					0.760	
	Parents					0.759	
	Children					0.749	

**Table 3:** Reliability and validity test.

Factors	Items	Standardized Factor Loading	SMC	CR	AVE	Cronbach Alpha
Behavior Attitude	BA1	0.917	0.841	0.924	0.755	0.777
	BA2	0.931	0.867			
	BA3	0.899	0.808			
	BA4	0.710	0.504			
Subjective Norm	SN1	0.919	0.845	0.946	0.855	0.867
	SN2	0.916	0.839			
	SN3	0.938	0.880			
Perceived Behavioral Control	PBC1	0.980	0.958	0.981	0.928	0.831
	PBC2	0.991	0.984			
	PBC3	0.984	0.970			
	PBC4	0.896	0.797			
behavior intention	BI1	0.954	0.910	0.971	0.894	0.866
	BI2	0.948	0.899			
	BI3	0.947	0.897			
	BI4	0.932	0.869			
Condition of the City Where the PHCI Is Located	Location	0.889	0.790	0.961	0.778	0.938
	Economics	0.903	0.815			
	Transportation	0.882	0.778			
	Culture and Customs	0.868	0.753			
	Development	0.905	0.828			
	Environment	0.885	0.792			
	Local reputation of the PHCI	0.833	0.689			
Remuneration and Development	Performance Assessment	0.894	0.801	0.967	0.788	0.945
	Working Subsidy	0.936	0.878			
	Holidays Arrangements	0.857	0.734			
	Working Bonus	0.911	0.832			
	Medical Insurance	0.938	0.884			
	Wage	0.921	0.846			
	Social Insurance and Housing Accumulation Fund	0.841	0.706			
	Position Promotion	0.789	0.624			
Internal Organization Development	Department Setting	0.886	0.787	0.976	0.758	0.961
	Specialist Construction	0.900	0.812			
	Knowledge Level	0.902	0.814			
	Human resource allocation	0.895	0.801			
	Regulatory regime	0.896	0.810			
	Teaching and scientific research	0.862	0.740			
	Learning Atmosphere	0.879	0.774			
	Hospital Culture	0.890	0.792			
	Personnel Quality	0.881	0.776			
	Services Scope	0.840	0.707			
	Software and Hardware Facilities	0.860	0.740			
	Leading and Administrative Capacity	0.803	0.645			
	Internal interpersonal relationship	0.809	0.656			
Job Responsibilities	Working Hours	0.796	0.634	0.9244	0.754	0.911
	Working Stress	0.894	0.801			
	Working Intensity	0.936	0.878			
	Workload	0.857	0.734			
Family Support	Children	0.911	0.832	0.9101	0.717	0.876
	Spouse	0.938	0.884			
	House	0.921	0.846			
	Parents	0.841	0.706			
Sense of Gain	Fulfilling Personal Value	0.789	0.624	0.9344	0.781	0.877
	Job-Related Well-being	0.886	0.787			
	Professional Pride	0.900	0.812			
	Basic Living Security	0.902	0.814			

**Table 4:** The effects of factors on the three core variables of TPB.

Path	Effect	Estimate	SE/Boot SE	C.R.	*p*
CCPL→ AB	Indirect	0.062	0.013	5.231	<0.001
CCPL→ SN	Indirect	0.029	0.007	4.143	<0.001
IOD → AB	Indirect	0.259	0.017	14.882	<0.001
IOD → SN	Indirect	0.034	0.008	4.250	<0.001
IOD → PBC	Indirect	0.237	0.036	6.583	<0.001
SG→ AB	Direct	0.330	0.023	11.971	<0.001
RD → AB	Direct	0.042	0.021	1.984	0.047
	Indirect	0.021	0.011	2.889	0.004
	Total	0.103	0.026	4.200	<0.001
RD → SN	Indirect	0.030	0.007	4.286	<0.001
FS→ SN	Direct	0.097	0.021	4.843	<0.001
JR → SN	Indirect	0.037	0.009	4.111	<0.001
JR → PBC	Direct	0.428	0.094	10.493	<0.001

## Data Availability

The data presented in this study are available on request from the corresponding author. The data are not publicly available due to personal privacy.
